# Immunohistochemistry for c-MET, HLA class II, and CD68 does not show prognostic value in early-stage classic Hodgkin lymphoma patients in the EORTC-GELA H9 intergroup randomised trial

**DOI:** 10.1371/journal.pone.0360080

**Published:** 2026-10-08

**Authors:** Rodrigo Martinez Alcala, Lydia Visser, Catherine Fortpied, Christophe Fermé, Michel Henry-Amar, Berthe Aleman, Anne Arens, Sidsel Juul, Amjad Hayat, Ida Hude Dragičević, Martin Hutchings, Paul Meijnders, Pim Mutsaers, Ewa Paszkiewicz-Kozik, Sára Rossetti, Anna Sureda, Charlotte Syrykh, Sanne Tonino, Wouter Plattel, Arjan Diepstra

**Affiliations:** 1 Pathology and Medical Biology, University Medical Center Groningen, University of Groningen, Groningen, the Netherlands; 2 European Organisation of Research and Treatment of Cancer (EORTC) Headquarters, Brussels, Belgium; 3 Hematology department, Gustave Roussy, Villejuif, France; 4 Centre de Traitement des Données du Cancéropôle Nord-Ouest, Centre François Baclesse, Caen, France; 5 Radiation Oncology, The Netherlands Cancer Institute, Amsterdam, The Netherlands; 6 Medical Imaging, Radboud university medical center, Nijmegen, The Netherlands; 7 Oncology, Copenhagen University Hospital - Rigshospitalet, Copenhagen, Denmark; 8 Department of Haematology, University Hospital Galway, Galway, Ireland; 9 Hematology, Clinical Hospital Centre Zagreb, Zagreb, Croatia; 10 Haematology, Copenhagen University Hospital - Rigshospitalet, Copenhagen, Denmark; 11 Department of Clinical Medicine, University of Copenhagen, Copenhagen, Denmark; 12 Radiation Oncology, Iridium Network, University of Antwerp, Antwerp, Belgium; 13 Hematology, Erasmus Medical Center Cancer Institute, University Medical Center, Rotterdam, The Netherlands; 14 Maria Skłodowska-Curie National Research Institute of Oncology, Warsaw, Poland; 15 Clinical Hematology Department, Institut Català d’Oncologia – L’Hospitalet, Institut d’Investigació Biomèdica de Bellvitge (IDIBELL), Universitat de Barcelona, Barcelona, Spain; 16 Department of Pathology, CHU of Toulouse, IUCT-Oncopole, Toulouse, France; 17 Amsterdam University Medical Center, Amsterdam, the Netherlands; 18 Hematology, University Medical Center Groningen, University of Groningen, Groningen, the Netherlands; Qatar University College of Medicine, QATAR

## Abstract

**Background:**

In classic Hodgkin lymphoma (cHL), identifying high-risk patients is crucial for tailored treatment. Clinical risk factors are useful, but there is room for improvement. Although many diagnostic tissue-based biomarkers have been described to correlate with prognosis, none have achieved the accuracy to be included into daily clinical practice.

**Methods:**

The most promising tissue biomarkers, c-MET, HLA class II, and CD68, were tested in early-stage cHL for prognostic validation, individually and in combination with each other and established clinical risk factors. All biomarkers were assessed by immunohistochemistry in diagnostic tissue of patients included in the EORTC-GELA H9 trial. A case-cohort design was used, with progression-free survival (PFS) as the primary endpoint (n = 257; 105 PFS events). Hazard ratios (HRs) were estimated using Cox regression models with Barlow weighting to account for the case-cohort structure.

**Results:**

c-MET expression was detected in the tumor cells in 67% (108/162) of evaluable cases, and cell-surface expression of HLA class II was observed in 51% (70/137), consistent with earlier studies. CD68 expression was scored as the percentage of tissue surface area stained, with scores above the median (9.4%) designated high. In univariate Cox regression analyses, the HRs for PFS were: 0.752 for c-MET (95% confidence interval (CI): 0.456–1.241); 0.854 for HLA class II (95% CI: 0.503–1.448); and 0.864 for CD68 (95% CI: 0.518–1.440). Since none of the biomarkers were significantly associated with PFS in univariate analysis, no further analyses combining the three biomarkers were performed.

**Conclusions:**

Using a rigorous statistical approach, we found that none of the three candidate biomarkers correlated with patient outcomes. While this lack of validation may be explained by trial-specific factors, such as risk-adapted treatment, the exclusion of advanced-stage patients, or challenges in scoring, these biomarkers provide insufficient prognostic value for implementation in clinical practice.

**Registration:**

NCT00005584 (https://clinicaltrials.gov/study/NCT00005584).

## Introduction

Classic Hodgkin lymphoma (cHL) stands out as a distinctive type of B-cell malignancy, characterized by unique clinical and biological traits. In cHL, the tumor microenvironment (TME) is dominated by a rich and immunosuppressive non-malignant immune infiltrate, while the malignant Hodgkin Reed-Sternberg (HRS) tumor cells make up less than 1% of the tumor mass. The immune infiltrate includes CD4 and CD8 T cells, and more variably, B cells, macrophages, dendritic cells, eosinophils, neutrophils, mast cells, and natural killer cells. This heterogenous cellular composition and the lymphoma architecture form the basis of the histological classification of cHL into four subtypes: nodular sclerosis (NS), lymphocyte-rich (LR), mixed cellularity (MC), and lymphocyte-depleted (LD) [1]. Infection with Epstein-Barr Virus (EBV), present in up to 25–30% of the cases, is an additional factor that shapes the TME composition by enhancing the immunomodulatory and pro-survival features of the HRS cells [2]. Latent EBV infection has been linked to a slightly adverse prognosis in patients over the age of 50 years [3].

Despite significant advances with standard first-line treatments, comprising chemotherapy, with or without radiotherapy, about 15% of cHL patients either fail to enter complete remission or relapse shortly after finishing treatment [4]. Current treatment strategies seek to reach higher complete response rates while reducing severe immediate and long-term treatment-related toxicities such as cardiac disease, pulmonary side-effects, infertility, and an increased risk of secondary cancers, among others. Reduction of these toxic effects has high priority since the majority of cHL patients are young adults. Clinical risk factors, encompassing disease stage, patient age, and others, have proven helpful in guiding treatment decisions [4]. In addition, early assessment of metabolic tumor response through [^18^F]FDG_PET scanning has been established as a helpful tool, allowing for tailored treatment adjustments [4,5]. However, even with these tools in use, the same 15% of patients either do not respond to initial treatment or encounter relapse [6–8], underscoring the need for better predictive biomarkers and treatment strategies.

Over the past decades, researchers have investigated a wide range of tissue-based biomarkers for cHL, identifying more than a hundred candidates with potential prognostic value. However, most of these studies aimed to evaluate the biological significance of a novel marker, but were not (entirely) corrected for clinical risk factors and therefore were not designed to ensure prognostic impact in a clinical setting. CD68 has gained the most attention among all these biomarkers since it was first described in 2010, and mainly identifies tumor-associated macrophages within the cHL microenvironment [9]. High numbers of CD68-positive cells are associated with a significant adverse prognosis, and this finding has been supported by several other studies that included correction for clinical risk factors [10–19]. However, several other publications have not found a significant correlation [20–27]. This contradiction may be related to technical reproducibility issues and challenges in standardization. In addition to high CD68, a lack of tumor cell markers c-MET or HLA class II was found to have significant adverse prognostic value independent of clinical risk factors [28,29]. These findings have not yet been replicated by other research groups or in other patient cohorts.

To validate CD68, c-MET, and HLA class II as prognostic biomarkers in a rigorously controlled setting, we evaluated diagnostic tissue samples from patients included in the multi-center randomized clinical trial EORTC-GELA H9 (protocol number 20982) [30,31]. In short, patients were randomly assigned to receive either six or four cycles of ABVD followed by Involved-Field Radiotherapy (IFRT) (6-ABVD-IFRT and 4-ABVD-IFRT), or four cycles of BEACOPP_baseline_ followed by IFRT (4-BEACOPP_baseline_-IFRT). We designed our biomarker study to assess the prognostic significance of each biomarker individually and in combination, aiming to develop a multiparameter risk classifier. For all three markers, antibodies, staining protocols, and scoring were similar to the initial scientific papers identifying prognostic impact [9,10,28,29].

## Materials and methods

### Patients and lymphoma tissue

Tissue samples embedded in paraffin were retrospectively collected from the EORTC-GELA H9 trial (protocol number 20982). The purpose of the randomized phase III trial was to compare the effectiveness of different regimens of combination chemotherapy with or without radiation therapy in treating HL patients (Fig 1). In this prospective controlled trial in clinical stage I and II (supradiaphragmatic) cHL patients, a distinction was made between patients with favorable clinical characteristics (H9-F) and patients with unfavorable clinical characteristics (H9-U). The flow diagrams for the H9-F and H9-U trials have been reported previously and are shown in S1 Fig and S2 Fig [30,31]. In H9-F, patients with complete response (CR or CR unconfirmed (CRu)) after 6x EBVP chemotherapy, standard radiotherapy treatment (36 Gy IFRT) was compared to 20 Gy IFRT and to no additional radiotherapy with the intent to reduce late toxicity. The primary endpoint was relapse-free survival, defined as the time from the date of assessment of complete response (CR or CRu) until relapse or death from any cause. In H9-U patients, 6x ABVD was compared to 4x ABVD and 4x BEACOPP baseline, followed by 30 Gy IFRT in all arms. The primary endpoint was event-free survival, defined as the time from registration until progression/relapse or death from any cause.

**Fig 1:**
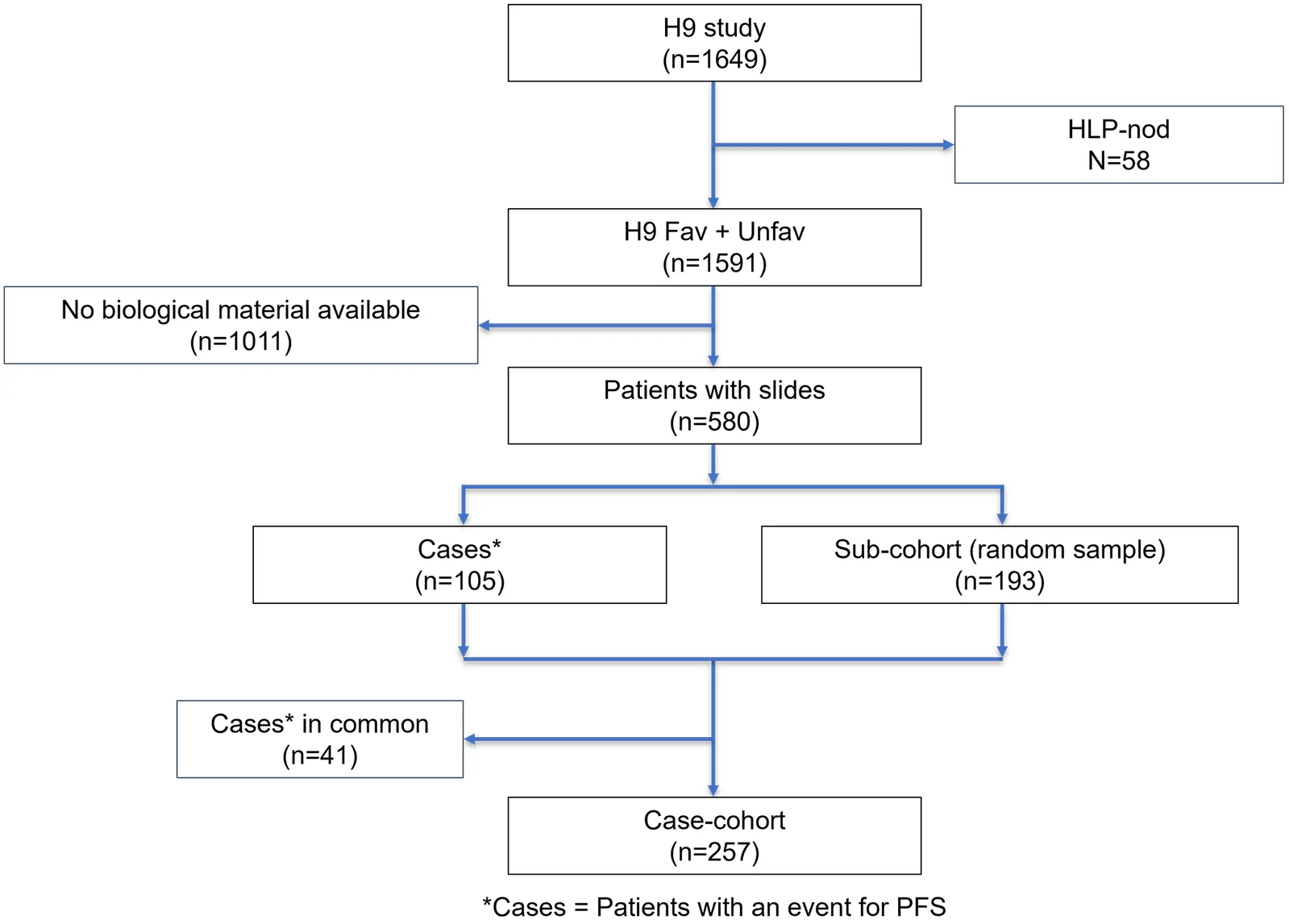
Flow diagram of patient samples used for this study. Cases are defined as patients who experienced an event for progression-free survival.

The H9 study included 1591 cHL patients enrolled between 01/10/1998 and 01/09/2002, and for 580 patients (36%), appropriate unstained whole tissue sections were available for analysis. These tissue sections were obtained from 50 centers in ten European countries: Belgium, Croatia, France, Italy, the Netherlands, Poland, Portugal, the Slovak Republic, Slovenia, and Switzerland. The trial was approved by the EORTC Protocol Review Committee and by ethics committees of each participating center or countries according to local laws. All patients gave written informed consent before study entry. The trial was registered at ClinicalTrials.gov under NCT00005584. Permission to conduct research on patient samples was obtained from the institutional review board of the University Medical Center Groningen (UMCG) (METc 2012.343). Data from patient samples were accessed for research purposes on 09/04/2020. Authors had no access to information that could identify individual participants during or after data collection. This study adheres to CONSORT principles by reporting key eligibility criteria, randomization procedures, and analysis population relevant to the evaluation of the collected patient samples^30,31^.

### Immunohistochemistry and in situ hybridization

Immunohistochemistry (IHC) was performed on consecutive slides of paraffin-embedded tissue sections. Depending on the origin of the samples, tissue sections were fixed with formalin or Bouin’s solution. Antibodies against the biomarkers c-MET, HLA class II, and CD68 were used. A rabbit polyclonal antibody to human antigen c-MET C-28 (RRID: AB_631941; dilution 1:200) was obtained from Santa Cruz Biotechnology (Cat. No sc-161). A mouse monoclonal antibody to human antigen HLA class II (clone CR3/43; RRID: AB_2313661; dilution 1:200) was obtained from Dako Denmark (Cat. No M0775). A mouse monoclonal antibody to human antigen CD68 (clone KP1; RRID: AB_2661840; dilution 1:2000) was obtained from Dako Denmark (Cat. No GA60961−2).

IHC for c-MET, HLA class II, and CD68 was performed as previously described [9,10,28,29]. Appropriate positive and negative controls were included for each staining. Fig 2 shows representative IHC staining images for the three biomarkers. To enable determination of tumor cell EBV status, Epstein-Barr virus-encoded small RNAs (EBER) in situ hybridization (ISH) was performed manually using the EBER RNA CISH kit (Dako Omnis).

**Fig 2:**
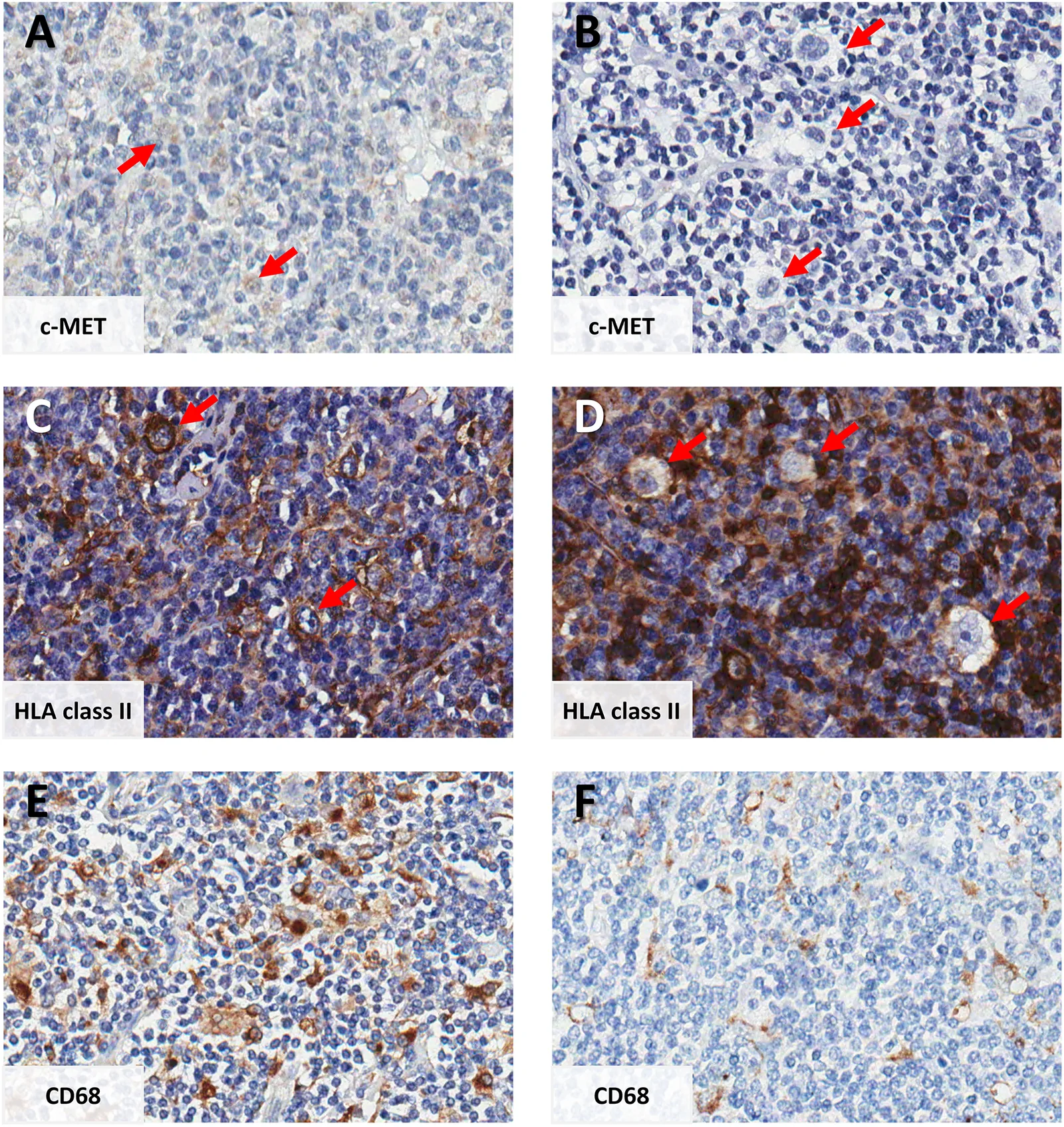
Representative immunohistochemistry of cHL tissue. For c-MET, panel (A) shows a case with expression in the cytoplasm of HRS cells, while panel (B) shows a patient with c-MET-negative HRS cells. For HLA class II, panel (C) shows a case with expression on the membrane of HRS cells and panel (D) displays a case with no expression. For CD68, panel (E) shows a case with >9.8% of surface area stained while panel (F) shows a case with ≤9.8% of positive surface area. Red arrows indicate HRS cells.

### Immunohistochemistry scoring

Stained slides for c-MET and HLA class II were analyzed by an experienced pathologist who also scored these stains in the original papers (A.D.), and CD68 stains were evaluated by automated digital scoring. The evaluation method for CD68 was selected to replicate the approach used in the original study, in which automated scoring was specifically performed in tumor-rich areas.

For c-MET, scoring of HRS cell staining was performed as previously reported [28]. Stained slides were considered suitable for evaluation only if at least ten HRS cells were present; otherwise, slides were classified as tissue with missing data. Cytoplasmic staining in at least 30% of the HRS cells was scored positive. This cut-off was based on a bimodal distribution of c-MET expression observed in the original study, where 30% of c-MET-positive tumor cells reflected two prognostically different groups.

HLA class II was scored using the same approach as originally described [29]. Stained slides were scored only when the positive control showed consistent staining of lymphocytes or sometimes vascular structures, and when at least 50 HRS cells were present for evaluation. Tissue sections that did not meet these criteria were considered unsuitable for scoring and classified as missing data. Membranous HLA class II expression was scored positive if it was more pronounced than in surrounding lymphocytes or present between adjacent HRS cells. Lack of membranous staining between adjacent HRS cells was denoted as negative. In heterogeneous staining between tumor cells, the predominant pattern was scored. Heterogeneous staining without a predominant pattern was categorized as indecisive.

Images of the CD68 staining were acquired by digital scanning (Aperio) and analyzed using Aperio Image Scope (version 11). Four tumor-rich areas (selected by L.V.) were selected for each case for morphometric analysis using a hue value of 0.1, a hue width of 0.5, and a color saturation threshold of 0.04. Cases were excluded and categorized as missing if there was an insufficient number of tumor cells to identify the staining reliably. For the remaining evaluable cases, the number of CD68-positive pixels was divided by the total tissue pixels and multiplied by 100 to calculate the percentage of positive pixels. The cut-off used to classify the expression of CD68 as high or low was based on the median value of positive pixels, consistent with the approach used in the original study with automated scoring [10].

### Statistical analysis

The statistical analysis methods were pre-defined before the immunohistochemistry and ISH were performed. The primary endpoint of this study was progression-free survival (PFS), defined as the time from registration until disease progression/relapse or death from any cause, as a compromise between the primary endpoints of H9-F and H9-U. Overall survival (OS) was the secondary endpoint.

A case-cohort design was used due to its flexibility, as the sampling is done irrespective of patient outcome [32]. Such a design combines the advantages of a cohort study with the efficiency of a case-control study. By definition, a case-cohort is composed of a random subcohort of patients from the overall population, irrespective of patient outcome, and supplemented with all cases. In our study, the case-cohort was constructed among the set of patients with diagnostic tissue available for biomarker measurement (n = 580), which included 105 cases, i.e., 105 patients who experienced an event for PFS. The random sub-cohort consisted of a random sample of 33% of the patients with biological material, stratified for risk group (favorable vs. unfavorable) and treatment arm. The fraction of 33% was chosen to ensure adequate power to confirm the magnitude of biomarker effects observed in previous studies [33]. The resulting random sub-cohort was composed of 193 patients, including 41 cases. The 64 cases not included in the random subcohort were added, resulting in a case-cohort of 257 patients, including 105 cases. Chi-square test for binary variables or the Kruskal-Wallis test for continuous variables were used to check whether the subset of patients with tissue available (580 patients) and the sub-cohort (193 patients) were representative of the overall study population (1591 patients). The distribution of biomarker values, the association between biomarkers, and clinical and demographic characteristics were described in the sub-cohort.

The prognostic value of the biomarkers was analyzed in the case-cohort population. The over-representation of cases in the case-cohort was adjusted in the statistical analysis using weighting methods. A separate Cox regression model was used to assess the prognostic value of each of the biomarkers on PFS, with case and control observations weighted by their inverse sampling probabilities using the Barlow weighting scheme [32, 34]. The estimated hazard ratios (HR) and their 95% confidence interval were provided, using robust variance estimation. The analysis was conducted separately for each risk group (favorable and unfavorable) and the whole group. An additional analysis was conducted, stratifying the primary model by treatment arm. Other weighting schemes for case and control observations were used as sensitivity analyses to evaluate the robustness of the results obtained with the primary model: Prentice (1986) and Self & Prentice (1988) [35,36].

With this design and the available number of patients and events, the statistical power to detect a HR of 2.0, 2.5, or 3.0 was 79%, 96% and 99% respectively, when analyzing favorable and unfavorable risk patients together. The calculations were based on the method of Cai et al.[37], assuming a significance level of 0.05 (two-sided) and a maximum imbalance between biomarker positive: negative of 71%: 29%.

## Results

### Patient and tumor characteristics

In the random sub-cohort (193 patients), 51% of patients were female, the median age was 31 years (range 15–69), 70% were stage II, 26% had B-symptoms, and 19% had bulky mediastinal disease. The basic characteristics of the sub-cohort are presented in Table 1. There were no significant differences between the patients included and excluded from the sub-cohort, indicating that it was representative of the total H9 population.

**Table 1:** Basic clinical characteristics of the total H9 population of cHL patients, the sub-cohort, and patients not included in the sub-cohort.

CHARACTERISTICS	TOTAL POPULATION		SUB-COHORT				*P-VALUE*
			NO		YES		
	N = 1591		N = 1398		N = 193		
Sex, no (%)							0.413
Male	827	(52)	732	(52)	95	(49)	
Female	764	(48)	666	(48)	98	(51)	
Age, median years (range)	30.6	(14.9–69.5)	30.6	(14.9–69.5)	30.5	(15.3–69.3)	0.956
Ann Arbor Stage, no (%)							0.710
I	460	(29)	402	(29)	58	(30)	
II	1131	(71)	996	(71)	135	(70)	
B-symptoms, no (%)							0.668
No	1109	(70)	976	(70)	133	(69)	
Yes	398	(25)	347	(25)	51	(26)	
Missing	84	(5)	75	(5)	9	(5)	
Bulky disease, no (%)							0.973
No (MT ratio < 0.35)	705	(44)	624	(45)	81	(42)	
Yes (MT ratio ≥0.35)	320	(20)	283	(20)	37	(19)	
Missing	566	(36)	491	(35)	75	(39)	

*P-values* are for comparing the sub-cohort and patients not included in the sub-cohort.

In the sub-cohort, 22% (38/174) of cases were EBV positive. Subtypes consisted of NS (84%) and the rest were either MC or LD (16%).

Median duration of follow-up was 7.8 years (inter-quartile range IQR = 5.7–9.3).

Although the case-cohort may have been potentially biased, its clinical characteristics were broadly comparable to those of the total H9 population. Detailed clinical data of the case-cohort are provided in S1 Table.

Binary variables were compared using Chi-square test, whereas continuous variables were compared using Kruskal-Wallis test. Patients with missing values were excluded from the statistical tests.

### Expression of biomarkers in the sub-cohort and the case-cohort

Positive expression of c-MET was 67% for both the sub-cohort and the case-cohort. The intensity of the staining of c-MET was generally weak but clearly recognizable due to low background staining. In the sub-cohort, data were missing for 16% of the patients, and in the case-cohort, for 17% of the patients. Missing data for c-MET were due to a lack of tissue for the staining, an insufficient number of tumor cells for reliable scoring, and failed staining.

HLA class II expression was positive in 51% of the sub-cohort and 52% of the case-cohort. Membranous staining of HLA class II on tumor cells was generally evident, appearing stronger than that of the surrounding lymphocytes or present between adjacent tumor cells. Scoring was considered indecisive in 9% of patients in the sub-cohort and 8% of patients in the case-cohort. Data were missing for 20% of patients in both the sub-cohort and the case-cohort. The reasons for missing data included: no available tissue, insufficient tumor cells for reliable scoring, and failed staining.

For CD68, automated digital image analysis was used to quantify expression, based on the proportion of positive pixels. The median proportion of CD68-positive pixels was 9.4% (range: 0.5–67.4) in the sub-cohort and 9.8% (range: 0.2–67.4) in the case-cohort. The staining intensity was generally strong and specific. Missing data were recorded for 22% patients in the sub-cohort and 23% patients in the case-cohort. Reasons for having missing data included: no available tissue, insufficient number of tumor cells to select tumor-rich areas, and failed staining. Detailed data for both groups are presented in Table 2.

**Table 2:** Biomarker scores in the sub-cohort and the case-cohort.

BIOMARKER	SUB-COHORT (N = 193)		CASE-COHORT (N = 257)	
c-MET, no (%)				
Negative	54	(33)	71	(33)
Positive	108	(67)	142	(67)
Missing	31	–	44	–
HLA class II, no (%)				
Negative	67	(49)	88	(48)
Positive	70	(51)	96	(52)
Indecisive	17	–	21	–
Missing	39	–	52	–
CD68, digital scoring of 4 tumor areas				
Median, % of positive pixels (range)	9.4	(0.5–67.4)	9.8	(0.2–67.4)
Missing, no (%)	42	–	60	–

### Patient survival

The primary analysis showed no statistically significant association between c-MET expression on tumor cells and PFS (HR: 0.752; 95% CI: 0.456–1.241). Similarly, there was no statistically significant association of membranous HLA class II expression (HR: 0.854; 95% CI: 0.503–1.448) and CD68 expression in the tumor microenvironment with PFS (HR: 0.864; 95% CI: 0.518–1.440). Data on the favorable and unfavorable subgroups separately are shown in Table 3. Given the lack of prognostic value of these biomarkers taken individually, we did not proceed to combine them. Estimated survival curves are presented in S3 Fig. Similar to PFS, there was no statistically significant association between OS and any of the biomarkers.

**Table 3:** Overview of Hazard Ratios for positivity of the biomarkers and occurrence of PFS (including Barlow weights).

PRIMARY ANALYSIS FOR PFS	FAVORABLE (N = 143)		UNFAVORABLE (N = 114)		FAVORABLE + UNFAVORABLE (N = 257)	
	HR	95% CI	HR	95% CI	HR	95% CI
c-MET	0.578	0.305-1.092	1.013	0.435-2.362	0.752	0.456-1.241
HLA class II	0.783	0.401-1.529	1.065	0.444-2.554	0.854	0.503-1.448
CD68 by automated digital scoring of 4 tumor areas (cut-off = 9.8%*)	0.790	0.414-1.507	1.257	0.525-3.006	0.864	0.518-1.440

HR indicates hazard ratio for positive versus negative levels; and CI, confidence interval.

Missing values were excluded, and for HLA-II, indecisive scores were also excluded from the analysis.

*: 9.8% is the median CD68 in the case-cohort, based on 193 non-missing values

## Discussion

Currently, no diagnostic tissue-based biomarkers are available for prognostic risk stratification in cHL in a clinical setting. In this study, we evaluated the prognostic value of three promising candidates in a well-defined early-stage clinical trial population: c-MET, HLA class II, and CD68. None of the biomarkers demonstrated independent prognostic value for PFS using a case-cohort design and a rigorous statistical methodology. These findings contrast with previous reports and emphasize the complexity of validating prognostic biomarkers in clinical trials. Besides the possibility of limited inherent prognostic relevance, several factors may explain the lack of validation, including aspects of study design and patient-specific factors, tissue quality, and statistical considerations.

Our cohort included only patients with early-stage cHL in the EORTC-GELA H9 trial. This focus allowed for a controlled analysis of treatment intensity and survival outcomes, but may have limited the variability needed to detect prognostic effects [30,31]. For example, c-MET expression was previously associated with worse outcomes in limited-stage disease. However, the statistical significance was lower (HR = 4.4) compared to the effect found in all stages (HR = 5.1) or advanced-stage patients (HR = 5.0) [28]. Although the H9 trial included patients at relatively high risk for an event (H9-U), the uniformly high survival outcomes in the H9 study may have reduced the possibility of detecting prognostic impact.

Both H9-U and H9-F trials reported excellent outcomes with 5-year event-free survival rates exceeding 85% in all treatment arms of the H9-U [30], and similarly high relapse-free survival rates in the H9-F [31], except for the arm omitting radiotherapy. Thus, effective treatment adaptation based on clinical risk factors may have created a ceiling effect that overrides biomarker-based prognostic differences.

The tissue samples were stained in a single center, but samples originated from 50 institutions across ten European countries, introducing variability in tissue processing and fixation. In contrast, original biomarker studies involved fewer centers, potentially limiting this variability in tissue sample characteristics. Variable tissue integrity may have compromised staining quality and scoring, particularly for c-MET, which has a low-intensity signal [28]; HLA class II, which shows diverse expression patterns in both HRS cells and TME [29]; and CD68, were scoring requires a well-defined cut-off for accurate assessment [9,10]. Eventually, some tissue sections were excluded due to poor quality, whichdiluted statistical power to some extent**.** Of note, we included EBER staining for descriptive purposes, but the number of positive cases was lower than expected. It was positive in only 22% of the sub-cohort, while ~30% would be expected [3].

The case-cohort design allowed efficient biomarker evaluation in a representative subset of the total study population. This approach was especially useful in evaluating three biomarkers, where procedures can become labor-intensive and time-consuming [38]. It is a flexible design that allows both description of the frequency of biomarker positivity in the patient population and evaluation of the prognostic effects of the biomarkers alongside clinical risk factors. We are confident that our approach is reliable because the clinical characteristics of the random sub-cohort were comparable to those of the overall H9 population, thus enabling generalizability of the findings. We applied the Barlow weighting scheme in the Cox regression model to account for over-representation of cases in the case-cohort, and as such to prevent bias when estimating the prognostic value of the three biomarkers. Other weighting schemes were also applied to check the robustness of the findings, and provided similar results. Finally, while biomarker selection was based on previously reported multivariate associations with clinical covariate adjustments, the supporting evidence for c-MET and HLA class II is limited, and CD68 results remain inconsistent across studies even when adjusting for clinical factors. These findings suggest that the prognostic value of these biomarkers may be context-dependent, influenced by age of the study population, disease stage, treatment regimen, methodology, and technical variability. Future research may benefit from exploring the predictive potential of these biomarkers and specifically considering their ability to identify patients who respond to a specific treatment regimen, rather than solely focusing on prognostic associations. Future research may benefit from prioritizing the predictive value of these biomarkers, which is increasingly more relevant than their prognostic role. In particular, their ability to identify patients who are likely to respond to specific, continuously evolving treatment regimens in cHL will be essential. Ideally, they should be re-validated early in clinical trials and subsequently confirmed in real-world clinical settings.

## Conclusions

In short, our results indicate that c-MET, HLA class II, and CD68 do not provide clinically relevant prognostic information in early-stage cHL. These findings highlight the importance of rigorous validation procedures even within the controlled setting of a clinical trial. They also underscore the importance of prospective integration, methodological rigor, and careful attention to biological, technical, and clinical variables that may influence biomarker performance. Our results pinpoint some of the factors that may limit the introduction of tissue-based biomarkers into clinical practice.

## Supporting information

S1 Table Clinical characteristics of the case-cohort. (DOCX)

S1 Fig CONSORT (Consolidated Standards of Reporting Trials) diagram for the H9-F trial. (TIF)

S2 Fig CONSORT (Consolidated Standards of Reporting Trials) diagram for the H9-U trial. (TIF)

S3 Fig Estimated Progression-Free Survival curves according to the expression of the tissue biomarkers. For the expression of biomarkers on HRS cells, neither (A) c-MET negativity nor (B) lack of expression of HLA class II is associated with increased survival. Positivity of >9.8% of CD68 on the tissue microenvironment is not associated with adverse survival for cases scored by (C) automated digital scoring of four tumor areas.
(TIF)
