# Supplementary material for: Immunohistochemistry for c-MET, HLA class II, and CD68 does not show prognostic value in early-stage classic Hodgkin lymphoma patients in the EORTC-GELA H9 intergroup randomised trial
Source: PLoS One. 2026 Oct 8;21(10):e0360080. doi: 10.1371/journal.pone.0360080 (PMC13648959; doi:10.1371/journal.pone.0360080)
Supplement: Supplementary file 1 [file pone.0360080.s001.docx]

# **Supplemental Material**

**S1 Table. Clinical characteristics of the case-cohort.**

| CHARACTERISTICS | CASE-COHORT | |
| --- | --- | --- |
|  | N=257 | |
| Sex, no (%) |  |  |
| Male | 129 | (50) |
| Female | 128 | (50) |
| Age, median years (range) | 31.3 | (15.3 – 69.3) |
| Cases for PFS, no (%) |  |  |
| PD / death | 105 | (41) |
| No PD / death | 152 | (59) |
| Ann Arbor Stage, no (%) |  |  |
| I | 74 | (29) |
| II | 183 | (71) |
| B-symptoms, no (%) |  |  |
| No | 181 | (70) |
| Yes | 67 | (26) |
| Missing | 9 | (4) |
| Bulky disease, no (%) |  |  |
| Not bulky (<0.35) | 115 | (45) |
| Bulky (≥0.35) | 47 | (18) |
| Missing | 95 | (37) |
| EBER, no (%) |  |  |
| Positive | 48 | (19) |
| Negative | 181 | (70) |
| Missing | 28 | (11) |
